# Dr. Ulysses Higgins Brown

**Published:** 1901-02

**Authors:** 


Dr. Ulysses Higgins Brown, of Syracuse, N.Y., died suddenly in the city of New York, whither he had gone on business, December 27, 1900, aged 49 years. He was a licentiate of the Medical Society of the County of Onondaga, 1878, a member of the Medical Society of the State of New York, and an ophthalmologist of distinction.

Dr. Robert V. K. Montfort, of Newburgh, N. Y., died in that city December 29, 1900, aged 65 years. He was an alumnus of Albany Medical College, '59, served as a medical officer during the civil war with distinction, was formerly health officer of Newburgh, and was a prominent educator, having served many years as clerk of the board of education and superintendent of public schools of Newburgh.

